# Robot-assisted radical prostatectomy with clipless intrafascial neurovascular bundle-sparing approach: surgical technique and one-year functional and oncologic outcomes

**DOI:** 10.1038/s41598-020-74513-y

**Published:** 2020-10-19

**Authors:** Tae Young Shin, Yong Seong Lee

**Affiliations:** Department of Urology, Hallym University Sacred Heart Hospital, Hallym University College of Medicine, Anyang, 14068 Republic of Korea

**Keywords:** Urological cancer, Cancer, Oncology, Urology

## Abstract

Various neurovascular bundle-sparing techniques have been introduced to maximize recovery of erectile function after robot-assisted radical prostatectomy (RARP). The clipless intrafascial neurovascular bundle-sparing technique aims to preserve periprostatic structures and neurovascular bundles as much as possible by avoiding clipping of the vascular pedicles. This study reports 1-year functional and oncologic outcomes and postoperative complications in 105 patients with intact preoperative erectile function who underwent a modified clipless intrafascial neurovascular bundle-sparing RARP. Intact erectile function was defined as score ≥ 21 on the Sexual Health Inventory for Men questionnaire or ability to have sexual intercourse. Median follow-up was 26.5 months (IQR 15.25–48). Postoperative erectile function recovery rates were 71.4%, 81.9%, 88.6%, 92.4%, and 94.3% at 1, 3, 6, 9, and 12 months, respectively. The rate of positive surgical margins was 16.2% overall and 11.8% in patients with stage pT2 disease. The biochemical recurrence rate was 6.7% overall. The modified clipless intrafascial neurovascular bundle-sparing technique is safe and feasible and can achieve excellent recovery of erectile function after RARP. Further large-scale prospective comparative studies are warranted.

## Introduction

Radical prostatectomy is arguably the most common treatment for localized prostate cancer. The goal of radical prostatectomy is to achieve complete cancer control while preserving urinary continence and sexual function. The neural pathways involved in male erectile function were first described by Walsh and Donker^1^. However, improvement of erectile function outcome after radical prostatectomy requires increased understanding of the anatomy of the responsible nerves. Robot-assisted radical prostatectomy (RARP), with its magnification of the operative field, allows for meticulous dissection of the periprostatic structures and preservation of the neurovascular bundles (NVBs). In efforts to preserve postoperative erectile function, various RARP surgical techniques have been studied^2–5^. RARP can achieve consistent functional and oncological outcomes with a low complication rate^6,7^. Nonetheless, erectile dysfunction after neurovascular bundle-sparing RARP remains a concern, with reported incidence rates ranging from 7.1 to 81.3% at 1-year follow-up^8,9^. Data from the Prostate Cancer Outcomes Study indicate that post-RARP erectile dysfunction significantly affects health-related quality of life in younger men by affecting everyday interactions with partners and perceptions of their own sexuality^10^.

After reviewing and employing various erectile function-preserving RARP surgical techniques in our practice, we developed a surgical technique that simultaneously uses athermal dissection and the clipless intrafascial neurovascular bundle-sparing technique. In this study, we describe this modified neurovascular bundle-sparing technique and evaluate its postoperative functional and oncologic outcomes.

## Materials and methods

### Study population and design

We retrospectively reviewed the electronic medical records of 218 patients with biopsy-proven prostate cancer who underwent treatment for prostate cancer at Hallym University Sacred Heart Hospital between January 2017 and December 2018. Among these patients, 175 who underwent RARP by a single experienced surgeon (> 800 RARPs) were eligible for study inclusion. Patient characteristics and perioperative and oncologic data were recorded. Inclusion criteria were as follows: (1) patient age ≤ 70 years; (2) intact preoperative erectile function, defined as a Sexual Health Inventory for Men (SHIM) questionnaire score ≥ 21; (3) localized low-risk prostate cancer with Gleason score ≤ 7 (cT1–2N0M0); (4) clipless intrafascial neurovascular bundle-sparing RARP; and (5) minimum 1-year follow-up. Exclusion criteria included insufficient data, neoadjuvant hormonal treatment, prior radiation therapy, and prior transurethral resection of the prostate; we also excluded six patients who were transferred to our institution after being diagnosed with prostate cancer in other hospitals. All patient underwent preoperative multiparametric magnetic resonance imaging (mpMRI). Finally, 105 patients were included for analysis.

The study was designed to evaluate functional and oncologic outcomes and the postoperative complications at 12 months. Data were collected in a customized database and analyzed. All methods were carried out in accordance with relevant guidelines and regulations. To evaluate the procedural learning curve, patients were divided into two subgroups based on order of treatment: early (patients 1–52) and late (patients 53–105). Study approval was obtained from the Hallym University Sacred Heart Hospital Ethics Committee (No. 2018-05-012). Written informed consent was obtained from all patients.

### Data collection and outcomes

The following patient data was obtained: age, body mass index (BMI), American Society of Anesthesiologist (ASA) score, prostate volume, prostate-specific antigen (PSA) level, Gleason score of prostate biopsy specimen, and D'Amico risk classification. Baseline erectile function was assessed using an abridged Korean version of the SHIM questionnaire^11^. Intraoperative variables included overall operative time, estimated blood loss, blood transfusion, nerve-sparing laterality, complications (intraoperative and postoperative), and pelvic lymph node dissection (PLND). Postoperative complications were recorded and evaluated using the Clavien–Dindo classification^12^. Postoperative erectile function was evaluated using the SHIM questionnaire at 1, 3, 6, 9, and 12 months after RARP. Recovery of postoperative erectile function was defined as score ≥ 21 on the SHIM questionnaire or the ability to have successful sexual intercourse. Pathologic variables included pathologic cancer stage, Gleason score, and positive surgical margins (PSMs). Biochemical recurrence (BCR) was defined according to the European Association of Urology guidelines as two consecutive PSA values ≥ 0.2 ng/mL^13^.

### Surgical techniques

All patients underwent transperitoneal RARP. Standard patient positioning and port placement have been described previously^14^. The da Vinci Xi Surgical System (Intuitive Surgical, Sunnyvale, CA, USA) was used in all cases.

**Figure 1:**
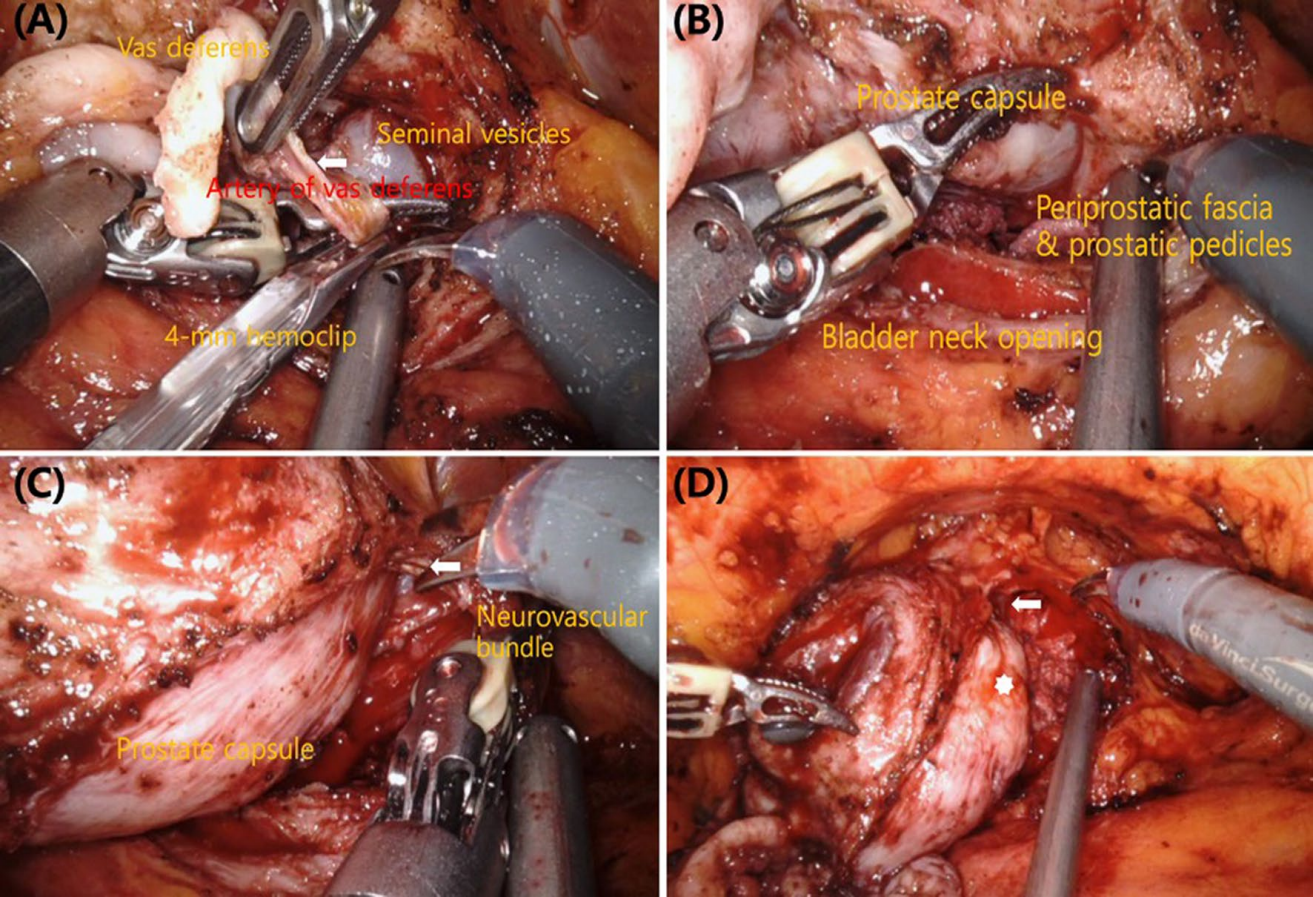
Operative steps. (**A**) The vas deferens and seminal vesicles were dissected athermally (artery of the vas deferens, white arrow). (**B**) The prostate capsule was exposed by detaching the overlying periprostatic fascia and prostatic pedicles. (**C**) The prostatic pedicles were further mobilized, including the neurovascular bundles, using antegrade dissection (distal end of the prostatic pedicles, white arrow). (**D**) Combined blunt and sharp dissection of the neurovascular bundles was performed as far distal to the apex as possible until the urethra was reached (prostate capsule, white star; urethra, white arrow).

**Figure 2:**
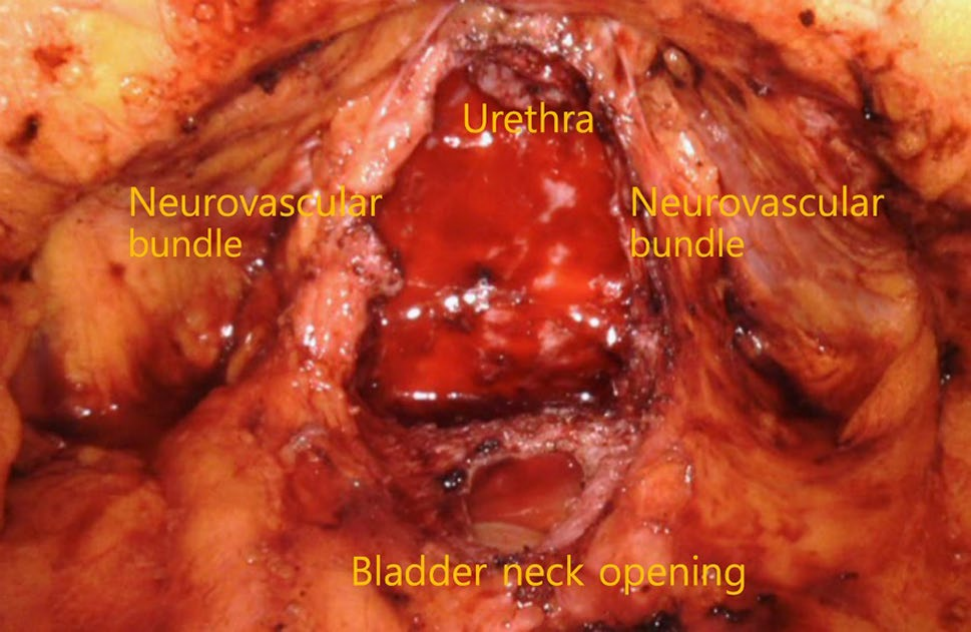
After complete prostate dissection, the preserved neurovascular bundles and prostatic pedicles are clearly visible and appear thick.

1. Athermal dissection of the vas deferens and seminal vesicles
  After bladder neck dissection, we performed athermal dissection of the vas deferens and seminal vesicles using Hem-o-Lok clips or 4 mm hemoclips to avoid thermal damage to the tissue; the use of monopolar electrocautery lateral to the seminal vesicles was avoided. The artery to the vas deferens, located between the seminal vesicles and the vas deferens, was resected *en bloc* (Fig. 1A).
2. Clipless intrafascial neurovascular bundle-sparing technique
  The endopelvic fascia was preserved in patients with clinical stage ≤ T2c disease. In patients with indications for clipless intrafascial neurovascular bundle-sparing, the robotic grasper arm was used to carefully expose the prostate capsule to detach the overlying fat and periprostatic fascia (Fig. 1B). Arterial pulsations from the cavernous vessels within the NVB were easily noted with further lateral dissection. These vessels were preserved by gently pushing them posterolaterally toward the rectum. The prostatic pedicles were further mobilized off the prostate capsule anteriorly to the most distal ends of the vascular pedicles (Fig. 1C). The identified vascular pedicles were then swept off the prostate, further mobilizing the NVBs, which were then gently eased off of the posterolateral surface of the prostate capsule with a combination of blunt and sharp dissection. We resumed antegrade dissection by peeling off the periprostatic fascia, NVBs, and prostatic pedicles *en bloc* until reaching the urethra (Fig. 1D). The use of electrocautery and clips during the nerve-sparing procedure was avoided. When bleeding from the periprostatic vessels occurred, brief pressure was applied using SURGICEL hemostatic gauze; mild venous bleeding was left uncontrolled. When pulsatile arterial bleeding was encountered, ligation was performed with 4–0 V-Loc suture. After the prostate dissection was complete, the preserved NVBs and prostatic pedicles were clearly visible and appeared thick (Fig. 2).
3. PLND
  PLND was performed in all patients. In patients with a > 5% risk of lymph node involvement in the Briganti nomogram, extended PLND to the common iliac artery area was performed^15^. Limited PLND to the obturator fossa was performed in those with estimated risk < 5%. Hem-o-Lok clips were used instead of cauterization during lymph node dissection to prevent lymphocele formation.

### Statistical analyses

Statistical analyses were conducted using SPSS Statistics for Windows version 26.0 (IBM Corp., Armonk, NY, USA). Continuous variables are reported as medians with interquartile range (IQR); categorical variables are reported as percentages. Erectile function was analyzed using the Kaplan–Meier method; its association with preoperative and intraoperative variables was tested using logistic regression analysis. Subgroup analyses were performed in patients with suspected posterolateral tumor on preoperative mpMRI. P < 0.05 was considered significant.

## Results

### Patient characteristics

Baseline demographic, clinical, and pathologic data for the patients are summarized in Table 1.

**Table 1:** Patients characteristics (N = 105).

	N = 105
Age, median (IQR), year	60.5 (52.25–69.0)
BMI, median (IQR), kg/m^2^	25.2 (23.8–28.0)
ASA score, median (IQR)	2.0 (1.0–2.0)
PSA, median (IQR), ng/ml	6.75 (3.2–11.5)
Prostate volume, median (IQR), cc	36.4 (20.5–76.0)
20–40	73 (69.5%)
41–60	20 (19.1%)
> 60	12 (11.4%)
**Biopsy Gleason score**	
6	11 (10.5%)
7	94 (89.5%)
**mpMRI site of tumor**	
Negative	9 (8.6%)
Apical	37 (35.2%)
Basal	7 (6.7%)
Posterolateral	30 (28.6%)
Anterior	6 (5.7%)
Multiple	16 (15.2%)
SHIM score, median (IQR)	22.5 (21–24)
≥ 21	105 (100%)
**D'Amico risk group**	
Low risk	30 (28.6%)
Intermediate risk	56 (53.3%)
High risk	19 (18.1%)

### Operative outcomes and complications

Median operative time was 210 min (IQR, 140–230). Median intraoperative blood loss was 200 mL (IQR, 150–450); only one patient with cardiovascular disease received an intraoperative blood transfusion. Pelvic lymphadenopathy was not found in any patient. Median length of hospital stay was 7 days (IQR, 7–7). Mean number of days with urinary catheter was seven (IQR, 7–7). The overall complication rate was < 10% (Table 2). No intraoperative complications occurred. Hematoma or lymphocele that required further procedures did not occur. At 12 months follow-up, no patients reported urinary retention or leakage.

**Table 2:** Intraoperative, histopathologic, and postoperative data (N = 105).

	N = 105
Operative time, median (IQR), min	210 (140–230)
Blood loss, median (IQR), ml	200 (150–450)
Blood transfusion	1 (1.0%)
**Neurovascular bundle-sparing**	
Bilateral	96 (91.4%)
Unilateral	6 (5.7%)
None	3 (2.9%)
**PLND**	105 (100%)
Extended PLND	10 (9.5%)
Limited PLND	95 (90.5%)
**Complications**	
Clavien grade 1	
Clavien grade 2	9 (8.6%)
Clavien grade 3	0
**Pathologic stage**	
pT2	93 (88.6%)
pT3a	8 (7.6%)
pT3b	4 (3.8%)
**Pathologic Gleason score**	
6	12 (11.4%)
7	87 (82.9%)
> 8	6 (5.7%)
**PSMs rate**	
Overall	17 (16.2%)
Among pT2 (93 patients)	11 (11.8%)
Among pT3 (12 patients)	6 (50.0%)
**PSMs site**	
Apical	9 (52.9%)
Posterolateral	5 (29.4%)
Basal	3 (17.7%)
Positive PLND	0

### Erectile function outcomes

**Figure 3:**
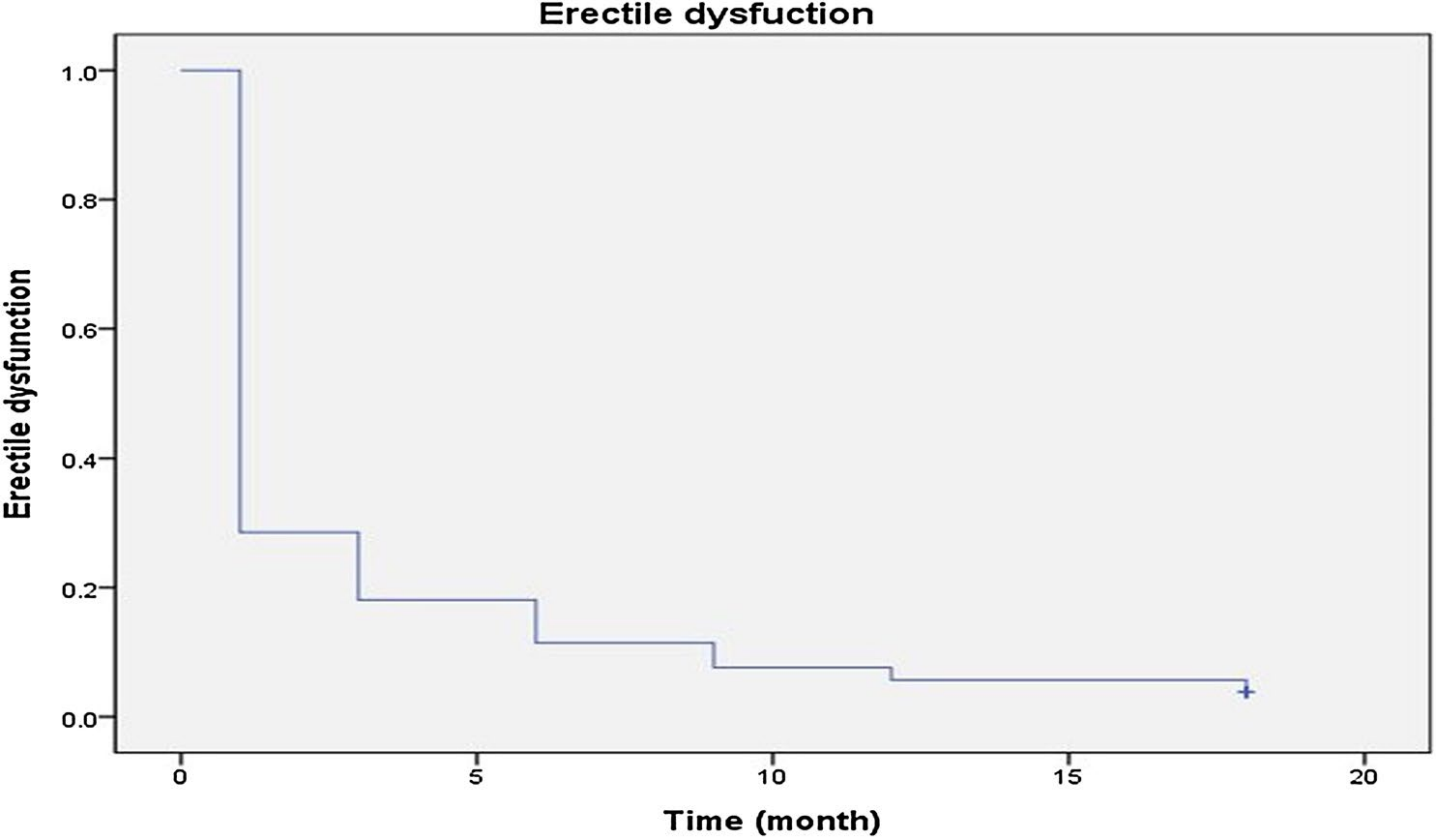
Kaplan–Meier curve showing recovery of erectile function over time.

All patients received penile rehabilitation with regular use of oral phosphodiesterase type 5 (PDE5) inhibitors from 1 week postoperatively until recovery of erectile function. Rates of recovery of erectile function were 71.4%, 81.9%, 88.6%, 92.4%, and 94.3% at 1, 3, 6, 9, and 12 months after RARP, respectively. Figure 3 shows the Kaplan–Meier curve of erectile function recovery. Median time to recovery was 21 days (IQR, 21–90). Regarding the learning curve analysis, progressive change in the number of patients with recovered function and operative time at each time point was not recorded. Of the 105 study patients, 86 (82%) experienced recovery of erectile function by 3 months; the remaining 19 could achieve a partial erection insufficient for penetration with use of PDE5 inhibitors.

Multivariate logistic regression analysis showed a significantly higher rate of erectile function recovery at 1 and 3 months after RARP with the bilateral clipless intrafascial neurovascular bundle-sparing approach (p < 0.001). Patients with low-risk D'Amico classification had a significantly higher rate of erectile function recovery at 1, 3, and 6 months (p < 0.05). However, at 6 months, the significant independent predictors were BMI, prostate volume, low or intermediate D'Amico risk classification, and bilateral neurovascular bundle-sparing approach (p < 0.05; Table 3).

**Table 3:** Multivariate analysis of erectile function outcomes within 6 months after clipless intrafascial neurovascular bundle-sparing robot-assisted radical prostatectomy.

	Erectile function at 1 month		Erectile function at 3 months		Erectile function at 6 months	
	OR (CI 95%)	p	OR (CI 95%)	p	OR (CI 95%)	p
Preoperative function†	2.155 (0.533–1.125)	0.350	0.905 (0.157–4.778)	0.865	0.675 (0.131–3.597)	0.620
BMI	0.955 (0.744–1.255)	0.115	2.655 (0.728–9.535)	0.065	1.728 (0.422–5.572)	0.028
Prostate volume	2.123 (0.155–5.442)	0.064	1.188 (0.082–3.866)	0.051	0.823 (0.338–1.597)	0.046
**D'Amico risk group**						
Low risk	1.242 (0.145–2.025)	0.025	1.824 (0.125–5.524)	0.038	0.952 (0.098–1.975)	0.082
Intermediate risk	1.185 (0.066–7.167)	0.092	1.552 (0.902–2.225)	0.168	1.034 (0.914–1.857)	0.045
High risk	0.907 (0.694–1.908)	0.362	1.113 (0.927–4.553)	0.471	1.143 (0.965–2.158)	0.628
**NVB-sparing**						
Bilateral	0.168 (0.053–0.566)	< 0.001	1.828 (0.178–3.325)	< 0.001	0.925 (0.092–1.452)	0.035
Unilateral	1.055 (0.056–5.623)	0.268	0.853 (0.657–2.326)	0.165	0.957 (0.769–1.081)	0.129

^†^Erectile function was defined as the Sexual Health Inventory for Men questionnaire score ≥ 21.

### Pathologic findings and oncological results

Table 2 shows the histopathologic data. Overall, 17 of 105 patients (16.2%) exhibited PSMs on postoperative pathology; PSMs were found in the apical and posterolateral margins in nine and five patients, respectively. In the cohort of patients with stage pT2 disease, the PSMs rate was lower (11.8%; 11 of the 93 patients). Subanalysis of 30 patients with suspected posterolateral tumor on preoperative mpMRI showed posterolateral PSMs in 5 (16.7%).

Median follow-up was 26.5 months (IQR, 15.25–48). BCR occurred in seven patients (6.7%). Median PSA level at the time of BCR was 0.3 ng/mL (IQR, 0.2–0.95). Patients with BCR underwent pelvic MRI, bone scintigraphy, and chest and abdominal computed tomography. Four patients were treated with luteinizing hormone-releasing hormone. The other three patients exhibited persistently elevated or increasing PSA levels without evidence of metastasis and were treated with salvage pelvic radiotherapy and androgen deprivation therapy.

## Discussion

RARP has been increasingly accepted as a surgical treatment option for younger and healthier patients with localized prostate cancer over the last decade^16,17^. RARP achieves consistent oncological outcomes and is associated with a low risk of complications^18,19^. Nevertheless, post-prostatectomy erectile dysfunction is a common adverse effect. Recent studies suggest that the course of NVBs is more complex than Walsh described previously^1^. Tewari et al. described that the prostate rests on a hammock-like nerve distribution, demonstrating that the NVBs are more a network of multiple finely dispersed nerves than a distinct structure^20,21^. Furthermore, Eichelberg et al. showed that only 46%–66% of all nerves are found in the classical posterolateral location in relation to the prostate, while 21%–29% are identified on the anterolateral surface^22^. Therefore, preservation of the surrounding periprostatic structures and NVBs should be attempted as much as possible. The mechanism of postoperative erectile function recovery is complex and not completely understood. However, it is universally accepted that preserving the original anatomical structures related to the prostate is the key to ensuring functional recovery. The intrafascial neurovascular bundle-sparing technique is important in determining functional outcomes.

Similar to Chien et al.^23^, we have been performing the athermal clipless intrafascial neurovascular bundle-sparing technique in an antegrade fashion. This approach is athermal, completely eliminating the use of monopolar electrocautery. In addition, we dissect the NVBs off the prostate in a medial to lateral direction without clipping the vascular pedicles. Although distinguishing the plane between the prostate and the periprostatic fascia where the pedicle enters can be challenging, eliminating bulk clipping of the pedicles is possible provided meticulous dissection of the pedicle vessels as they enter the prostate. We believe that eliminating the use of electrocautery and bulk clipping of the prostatic pedicles may provide better tissue preservation with the NVBs during intrafascial neurovascular bundle-sparing RARP (Supplementary Video S1).

The risk for PSMs is certainly possible when approaching the posterolateral plane using the clipless intrafascial neurovascular bundle-sparing technique. However, this technique did not appear to affect oncologic outcomes in this study. Although our overall rate of PSMs (16.2%) was higher than that noted in a previously reported study^24^, our rate in the cohort of patients with pT2 stage disease (11.8%) was similar to or lower than that noted in another RARP study^25^. In our subanalysis of 30 patients with suspected posterolateral tumor on preoperative mpMRI, posterolateral PSMs were noted in 16.7%; thus, the clipless intrafascial technique does not pose a high risk of PSMs in patients with a posterolateral tumor.

The postoperative functional outcomes in this study are promising. The clipless intrafascial neurovascular bundle-sparing technique aims to accomplish an early return to erectile function via maximal preservation of the periprostatic anatomical structures in the pelvic cavity, which is associated with functional normalcy. Using a validated sexual function SHIM questionnaire at 1 month after RARP, 71.4% of our patients returned to their baseline preoperative sexual function scores, which increased to 81.9%, 88.6%, 92.4%, and 94.3% at 3, 6, 9, and 12 months, respectively. These recovery rates are favorable compared with a previous RARP study that used the same questionnaire (53.1%, 69.9%, 82.3%, and 86.7% at 1, 3, 6, and 12 months, respectively)^26^. Although the surgical techniques used in the clipless intrafascial neurovascular bundle-sparing approach are not particularly novel, we found that their combined use can result in excellent postoperative functional outcomes.

Several limitations of this study include its retrospective nature, non-comparative design, and small sample size. In addition, all surgeries were performed by a single surgeon at a single institution; the surgeon’s extensive experience may have influenced functional outcomes. Nonetheless, our initial results suggest excellent erectile function recovery after antegrade clipless intrafascial neurovascular bundle-sparing RARP. A long-term large-scale multicenter study comparing erectile function after standard and clipless intrafascial neurovascular bundle-sparing RARP is necessary to validate our findings.

## Conclusion

In summary, the clipless intrafascial neurovascular bundle-sparing technique is safe and feasible and can achieve excellent recovery of postoperative erectile function without compromising oncologic outcomes after RARP. Further comparative prospective studies are warranted.

## Supplementary information

Supplementary Video S1.
